# Clinicopathological Characteristics of Kikuchi-Fujimoto Disease: A Retrospective Case Series from Pakistan

**DOI:** 10.7759/cureus.110059

**Published:** 2026-06-01

**Authors:** Hanzla Maryam, Seemal Aslam, Muhammad Awais Kanwal, Salma Abbas, Tariq Mehmood, Muhammad Fasial, Akifa Abbas, Faisal Sultan

**Affiliations:** 1 Pathology, Shaukat Khanum Memorial Cancer Hospital and Research Centre, Lahore, PAK; 2 Infectious Diseases, Shaukat Khanum Memorial Cancer Hospital and Research Centre, Lahore, PAK; 3 Surgery, Shaukat Khanum Memorial Cancer Hospital and Research Centre, Lahore, PAK; 4 Medicine, Shaukat Khanum Memorial Cancer Hospital and Research Centre, Lahore, PAK

**Keywords:** case series, histiocytic necrotizing lymphadenitis, kikuchi fujimoto, lymphadenopathy, self-limiting, south asia

## Abstract

Background

Kikuchi-Fujimoto disease (KFD), also known as histiocytic necrotising lymphadenitis, is a rare, benign, and usually self-limiting condition that commonly presents with cervical lymphadenopathy, fever, and constitutional symptoms. It is most often reported in young Asian adults. Its aetiology remains unclear, and histopathological examination is essential to confirm the diagnosis and exclude important mimics such as lymphoma and lupus lymphadenitis.

Methods

This retrospective case series included 10 patients with histopathologically confirmed KFD who were diagnosed and managed at Shaukat Khanum Memorial Cancer Hospital and Research Centre between January 2000 and January 2024. Clinical, pathological, and laboratory data were collected and analysed.

Results

Among the 10 patients, the mean age was 26.3 ± 7 years. Eight were female patients, and two were male patients, resulting in a female-to-male ratio of 4:1. Cervical lymphadenopathy was the most common presentation. Fever was reported in six patients (60%), while weight loss and myalgia/arthralgia were each reported in two patients (20%). Two patients had lymphoid malignancies in remission. No autoimmune diseases were documented, although antinuclear antibody (ANA) testing was not performed routinely.

Conclusion

This case series highlights the varied clinical presentation of KFD and reinforces the importance of histopathological diagnosis in patients presenting with lymphadenopathy. Larger prospective studies are needed to better understand its associations and outcomes.

## Introduction

Kikuchi-Fujimoto disease (KFD), first described in Japan in 1972, is a rare, benign, and usually self-limiting form of histiocytic necrotising lymphadenitis. It predominantly affects young adults, particularly those of Asian descent [1,2]. It most commonly presents with cervical lymphadenopathy, often accompanied by systemic symptoms such as fever, malaise, night sweats or weight loss [3,4]. Although KFD has now been reported worldwide, its exact aetiology remains unclear, with infectious and autoimmune mechanisms frequently proposed [5-7].

Diagnosis is based on histopathological examination of an affected lymph node. Typical findings include paracortical necrosis, karyorrhectic debris, histiocytic infiltration and an absence of neutrophils [8]. These features are important in distinguishing KFD from conditions such as non-Hodgkin lymphoma, tuberculosis, and lupus lymphadenitis [9].

We report a retrospective case series of 10 patients with histopathologically confirmed KFD diagnosed and managed at our institution. This study describes their clinical, laboratory and pathological features, and adds regional data from Pakistan to the existing literature on this uncommon disease.

## Materials and methods

Study design and setting

This retrospective case series was conducted at the Shaukat Khanum Memorial Cancer Hospital and Research Centre (SKMCH&RC), Lahore, Pakistan. It included 10 patients diagnosed with KFD between January 2000 and January 2024. Patients were identified through the institutional pathology database, and the diagnosis was confirmed on histopathological examination of excisional lymph node biopsy specimens.

The aim of this study was to describe the clinical features, laboratory findings, histopathological characteristics, management and outcomes of patients with KFD, and to add regional data from Pakistan to the limited published literature on this rare and often under-recognised condition.

Inclusion and exclusion criteria

Patients were included if they had a definitive histopathological diagnosis of KFD and available clinical records. Patients were excluded if the histopathological diagnosis was not consistent with KFD, if an alternative diagnosis was established, or if the available clinical documentation was insufficient for meaningful analysis.

Data collection

Data were extracted from electronic medical records. Collected variables included age, sex, site of lymphadenopathy, presenting symptoms, duration of illness, associated conditions, laboratory findings, treatment, and follow-up outcomes.

Laboratory parameters included complete blood count, erythrocyte sedimentation rate (ESR), liver function tests, and autoimmune or infectious screening, where available. Histopathology reports were reviewed for features consistent with KFD, including necrotising lymphadenitis, karyorrhectic debris, histiocytic infiltration, and absence of neutrophilic infiltration. Immunohistochemistry findings were recorded where available.

Treatment was classified as symptomatic management, observation, treatment for associated infection, or other documented management. Outcomes were assessed using the available follow-up records and included clinical improvement, recurrence where documented, or loss to follow-up.

Ethical considerations

Ethical approval was obtained from the Institutional Review Board of Shaukat Khanum Memorial Cancer Hospital and Research Centre. The study was conducted in accordance with the principles of the Declaration of Helsinki. All patient data were anonymised before analysis, and no identifiable information was used in reporting.

## Results

Among the 10 patients (Table 1), eight were female subjects, and two were male subjects, yielding a female-to-male ratio of 4:1.

**Table 1:** Demographics and clinical profiles of the patients with Kikuchi-Fujimoto disease TLC: Total leucocyte count; ANC: Absolute neutrophil count; Hb: Hemoglobin; ALT: Alanine transaminase; AST: Aspartate transaminase; ESR: Erythrocyte sedimentation rate.

Characteristics	Case 1	Case 2	Case 3	Case 4	Case 5	Case 6	Case 7	Case 8	Case 9	Case 10
Demographics										
Age	22	20	22	14	22	34	32	37	27	33
Gender	Male	Female	Female	Female	Female	Female	Female	Female	Female	Male
Lymphadenitis										
Site	Cervical Supraclavicular	Cervical Axilla Inguinal	Supraclavicular	Cervical	Cervical	Cervical	Cervical	Cervical	Cervical pre- and post-auricular	Cervical
Clinical Presentation										
Fever	Yes	Yes	No	Yes	Yes	No	Yes	No	No	Yes
Myalgia/Arthralgia	No	Yes	No	No	No	No	Yes	No	No	No
Sore throat	No	No	No	Yes	No	No	No	No	No	No
Rash	No	No	No	No	No	No	No	No	No	No
Fatigue	No	Yes	No	No	No	No	No	No	No	No
Neurological symptoms	No	No	No	No	No	No	No	No	No	No
Weight loss	No	Yes	No	No	Yes	No	No	No	No	No
Night sweats	No	Yes	No	No	No	No	No	No	No	No
Associated conditions										
Autoimmune disease	No	No	No	No	No	No	No	No	No	No
Viral infection workup	-	-	-	Monospot +ve	-	-	-	-	-	-
Malignancy	No	No	No	No	No	No	DLBCL In remission	No	CHL In remission	No
Laboratory parameters										
TLC (/ul)	3.55	6.48	-	2.86	-	3.89	7.24	4.52	6.05	6.54
ANC (/ul)	1.8	3.15	-	1.11	-	1.92	3.37	1.71	4.02	4.2
Platelets (/ul)	195	218	-	206	-	383	291	316	348	231
Hb (g/dl)	14.5	12.8	-	9.9	-	10.9	12.3	12.6	12.8	11.7
ALT (IU/L)	38	10	-	12	-	-	-	-	-	166
AST (IU/L)	37	22	-	27	-	-	-	-	-	109
Total bilirubin (mg/dl)	0.74	0.26	-	0.54	-	-	-	-	-	2.25
ESR	-	65	-	40	-	-	74	-	67	46
Treatment given	Symptomatic	Symptomatic	-	Symptomatic	Symptomatic	-	Symptomatic	-	Symptomatic	Treated for concomitant bacteraemia
Follow- up / Last known status	Lost	Improved	Lost	Improved	Lost	Lost	Improved	Lost	Improved	Improved

The mean age was 26.3 ± 7 years, with the majority (70%) over the age of 20. Cervical lymphadenopathy was the most frequently observed site (90%), with supraclavicular and axillary nodes less commonly involved.

The most common symptom was fever, reported in six patients (60%), followed by weight loss and myalgia/arthralgia, each reported in two patients (20%). Other symptoms included sore throat, fatigue and night sweats, each reported in one patient (10%). No patients had documented rash or neurological symptoms. The duration of illness ranged from 0.5 to 28 months.

No patient had a known autoimmune condition. Antinuclear antibody (ANA) testing was not performed routinely, so an autoimmune association could not be fully excluded. One patient had a positive Monospot test, suggesting possible recent Epstein-Barr virus exposure, although further viral testing was not available. Two patients had lymphoid malignancies in remission, one with diffuse large B-cell lymphoma (DLBCL) and one with classical Hodgkin lymphoma (CHL). 

Laboratory findings varied across the cohort. Some patients had leukopenia, while ESR was elevated in all patients in whom it was tested. Management was mainly symptomatic. One patient received additional treatment for concomitant bacteraemia. Five patients showed clinical improvement during follow-up, while the remaining five were lost to follow-up.

For broader context, Table 2 presents a comparative analysis of clinical features from our series against other published studies.

**Table 2:** Comparative overview of clinical features across four studies EBV: Epstein-Barr virus; ESR: erythrocyte sedimentation rate. [9-11]

Characteristics	Our 10 patients	Hye-young kim et all (Sample size 80)	Abdel rahman al manasra et all (Sample size 11)	R A Abeysekera et all (Sample size 9)
Mean age	26.3 ± 7	21.5 ± 11.8	32 ± 27.5	21 ± 9
Female to male ratio	4:1	1.1 : 1	8:3	All females
Cervical lymphadenopathy	90%	90%	82%	88.80%
Fever	60%	65%	63%	100%
Myalgia / arthralgia	20%	13%	36%	Not mentioned
Rash	0%	7.50%	Not mentioned	0%
Sore throat	10%	Not mentioned	Not mentioned	Not mentioned
Fatigue	10%	Not mentioned	82%	Not mentioned
Neurological symptoms	0%	Not mentioned	Not mentioned	Not mentioned
Weight loss	20%	8.70%	27%	Not mentioned
Night sweats	10%	Not mentioned	9%	Not mentioned
Ana positive	Not checked	27.30%	18%	0% (Checked in 2)
EBV positive	Monospot +ve in 1 Not checked in others	16.70%	Not mentioned	Not mentioned
Malignancy	30%	Not mentioned	9%	0%
Leucocytosis	0%	0%	63%	0%
Elevated ESR	100% (checked in 5 patient)	73.50%	72%	55.50%
Relapsed	No relapse in 5 patients 5 lost to follow	11.80%	Not mentioned	0%

## Discussion

This case series provides a clinical and pathological review of 10 patients with KFD diagnosed and managed at our institution. Our findings are broadly consistent with the existing literature, showing that KFD commonly affects young adults, with a female predominance. In our cohort, the mean age was 26.3 years, and the female-to-male ratio was 4:1. This was in keeping with previous studies, including those by Kucukardali et al. and Seo et al., which also reported a higher frequency of KFD among young female patients [6,4].

The most common clinical presentation in our series was cervical lymphadenopathy, seen in 90% of patients. Fever was reported in 60% of patients, while myalgia/arthralgia and weight loss were each reported in 20%. These findings are broadly similar to those of previous published series, although symptoms such as rash, sore throat, and night sweats were less frequent in our cohort [9-11]. This variation may reflect the small sample size, differences in regional presentation, or incomplete symptom documentation due to the retrospective nature of the study.

No patients had a documented autoimmune condition. However, ANA testing was not performed routinely, so an autoimmune association cannot be fully excluded. This is important because KFD has a recognised clinical and histological overlap with systemic lupus erythematosus (SLE), and autoimmune screening may be useful in selected or clinically ambiguous cases. One patient had a positive Monospot test, suggesting possible recent Epstein-Barr virus exposure, although further viral testing was not done. Therefore, no definite infectious trigger could be established in this cohort.

Two patients had lymphoid malignancies in remission: one with DLBCL and one with CHL. One further patient had a benign pancreatic mass. The association between KFD and malignancy remains unclear and is not well established. In our cohort, these findings should be interpreted cautiously, particularly because the lymphoid malignancies were in remission and the pancreatic lesion was benign. However, the presence of previous lymphoid malignancy highlights the diagnostic importance of histopathological confirmation, as KFD can clinically mimic malignant lymphadenopathy.

Histologically, the cases demonstrated features consistent with KFD, including necrotising lymphadenitis, paracortical necrosis, karyorrhectic debris and histiocytic infiltration. The absence of neutrophils, eosinophils and granulomas supported the diagnosis and helped distinguish KFD from important mimics, such as lymphoma, tuberculosis and lupus lymphadenitis. Immunohistochemistry, when performed, helped support the diagnosis and exclude lymphoid malignancy [12-14].

This study reinforces the importance of clinicopathological correlation in patients presenting with lymphadenopathy. Although KFD is usually benign and self-limiting, it can be mistaken for lymphoma, tuberculosis or autoimmune lymphadenitis. Accurate recognition is therefore important to avoid unnecessary treatment, repeated investigations or patient anxiety. In settings where tuberculosis and lymphoma are common diagnostic considerations, awareness of KFD is particularly important.

The strengths of this study include histopathological confirmation in all cases, detailed clinical and pathological documentation. As the only series from Pakistan, this study adds useful regional data to the existing literature on KFD.

The main limitations are the small sample size, retrospective design, single-centre setting, incomplete autoimmune and infectious work-up, and loss to follow-up in half of the patients. The long study period may also have introduced variation in diagnostic work-up, pathology reporting and follow-up practices over time. These limitations mean that the findings should be interpreted cautiously and may not be generalisable to all patient populations.

## Conclusions

KFD should be considered in young patients presenting with lymphadenopathy and systemic symptoms, particularly when lymphoma, tuberculosis or lupus lymphadenitis are part of the differential diagnosis. Histopathological examination remains essential for confirming the diagnosis and avoiding misdiagnosis or overtreatment. This case series adds regional data from Pakistan and supports the need for larger prospective studies with complete autoimmune and infectious work-up, as well as longer follow-up, to better understand the associations and outcomes of KFD.
